# Identification of a small molecule 0390 as a potent antimicrobial agent to combat antibiotic-resistant *Escherichia coli*

**DOI:** 10.3389/fmicb.2022.1078318

**Published:** 2022-12-15

**Authors:** Linhui Li, Pengfei She, Shasha Liu, Yimin Li, Zehao Li, Yifan Yang, Linying Zhou, Yong Wu

**Affiliations:** ^1^Department of Laboratory Medicine, The Third Xiangya Hospital of Central South University, Changsha, China; ^2^Department of Laboratory Medicine, The Affiliated Changsha Hospital of Xiangya School of Medicine, Central South University, Changsha, China

**Keywords:** small molecules, antibiotic combination, antibacterial, polymyxin, *Escherichia coli*

## Abstract

**Introduction:**

Antibiotic resistance has posed a serious challenge to global public health. With the increasing resistance emergence of E. coli and mortality caused by drug-resistant E. coli infections, it is urgent to develop novel antibiotics.

**Methods:**

By high-throughput screening assay, we found a bioactive molecule, 0390 (6056–0390), which demonstrated antimicrobial effects against E. coli. The antimicrobial effects of 0390 alone or in combination with conventional antibiotics were assessed by scanning electron microscopy, transmission electron microscopy, drug combination assay, and growth inhibition assay. In addition, we investigated the antimicrobial efficacy in subcutaneous infection model in vivo

**Results:**

0390 showed significant synergistic antimicrobial effects in combination with SPR741, a polymyxin B derivative, against E. coli standard strain and extensively drug-resistant (XDR) clinical isolates, and the combination exhibited good safety property in vitro. In addition, we demonstrated that the combinational treatment of 0390 and SPR741 exhibited a considerable antibacterial activity in vivo, and no tissue damage or other toxicity was observed after the therapeutic dose treatment.

**Discussion:**

To confront the issue of the infectious diseases related to E. coli and its multidrug resistant strains, potential approaches, such as new antibacterial agents with different structures from conventional antibiotics and drug combinations, are urgently needed. In this study, we have determined the in vitro and in vivo antimicrobial potential of 0390 alone or in combination with SPR741, which might be used as a treatment option for E. coli related infections.

## Introduction

*Escherichia coli* (*E. coli*) is a Gram-negative, facultative anaerobic short bacillus, which was first found and isolated by Theodor Escherich in 1885 (Escherich, 1988). *Escherichia coli* is known as both harmless commensal of the gastrointestinal tract in warm-blooded animals and one of the most important pathogens in humans, which is the most frequent cause of urinary tract infections (Kaper et al., 2004; Weiner-Lastinger et al., 2020). These infections are common, but also associated with high morbidity and high mortality (Russo and Johnson, 2003; Lefort et al., 2011; Abernethy et al., 2015; Basmaci et al., 2015).

However, misuse and overuse of antibiotics are the main reasons for the emergence and spread of severe antimicrobial resistance and multidrug resistance (MDR; Llor and Bjerrum, 2014; Harkins et al., 2017). At Global Burden of Diseases, Injuries, and Risk Factors Study (GBD) 2019, antimicrobial resistance was the third leading GBD Level 3 cause of death (Antimicrobial Resistance Collaborators, 2022). Antibiotic-resistant bacteria are a threat to public health care and have triggered the development of global action plans (World Health Organization, 2015). The World Health Organization has included extended-spectrum β-lactamase (ESBL)-producing *E. coli* in the “Global Priority List of Antibiotic-resistant Bacteria to Guide Research, Discovery and Development of New Antibiotics” (World Health Organization, 2017). In 2019, six pathogens, *E. coli, S. aureus, K. pneumoniae, S. pneumoniae, A. baumannii,* and *P. aeruginosa*, led to more than 250,000 deaths related to antimicrobial resistance, and *E. coli* was ranked first for the amount of deaths (Antimicrobial Resistance Collaborators, 2022). It is urgent to develop new antibacterial agents to combat diseases related to *E. coli* and its resistant strains.

Confronting with the issue of antibiotic resistance, drug combination is also one of the potential approaches. Antibiotic adjuvants are compounds with weak antibacterial activity when they are used solely, but they can restore or potentiate the activity of antibiotic, such as SPR741 (Liu et al., 2019). SPR741 (NAB741) is a cationic peptide derived from polymyxin B and retains the ability to permeabilize the outer membrane of Gram-negative bacteria (Vaara et al., 2010). Polymyxins (polymyxin B and colistin) are often the only alternative for some pan-resistant Gram-negative bacteria, although the usage of polymyxins is shadowed by their toxicity, which is mainly referred to nephrotoxicity (Arias and Murray, 2009). Compared with polymyxin B, SPR741 exhibits minimal intrinsic Gram-negative antibacterial activity, but an excellent safety profile *in vivo*. Eckburg et al. (2019) has evaluated the safety, tolerability, and pharmacokinetics of SPR741 in humans. SPR741 is reported to combat Gram-negative bacteria in combination with traditional antibiotics, such as azithromycin and temocillin (Mendes et al., 2020a,b). However, the *in vitro* and *in vivo* effects of new identified antimicrobials in combination with SPR741 have not yet been reported.

Here, to identify novel antibacterial compounds with different structures from the conventional antibiotics, we screened MINI Scaffold Library (TopScience, L5600) containing 5,033 bioactive small-molecules and determined a novel small molecular compound, 0390, which was found effective alone or in combination with SPR741 to combat *E. coli*, including its standard strain and drug-resistant clinical isolates. In addition, the synergistic antimicrobial effects are observed in the subcutaneous abscess model, which was established by *E. coli* infection, while the combination treatment showed good safety property to the host. In this study, we conducted a series of *in vitro* and *in vivo* assays to provide an alternative regimen to treat *E. coli* related infections.

## Materials and methods

### Bacterial strains and culture conditions

The strains used in this study are listed in Supplementary Table S1. XDR *E. coli* Y0064, Y9395, Y9592, and Y9633 were kindly provided by Min Li (Shanghai Jiaotong University, Shanghai, China). Gram-positive cocci *Staphylococcus* was grown in tryptic soy broth (TSB; Solarbio, Shanghai, China), and *Enterococcus* was grown in brain-heart infusion (BHI; Solarbio, Shanghai, China). Gram-negative species were grown in Lysogeny Broth (LB; Solarbio, Shanghai, China). All bacteria were grown at 37°C and constantly shaking (180 rpm). All drugs used in this study were dissolved in deionized water or dimethyl sulfoxide (DMSO).

### High-throughput screening of compounds

**Figure 1:**
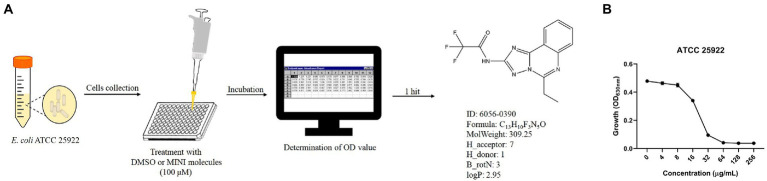
Antimicrobial effects of 0390 against *Escherichia coli*. **(A)** Workflow of the *in vitro* high-throughput screening assay of the MINI Scaffold library, containing 5,033 MINI molecules. The OD value was determined by a microplate spectrophotometer. The structural formula and characteristics of a hit (hit compound), 0390 (6056–0390) were shown. H_acceptor: hydrogen bond acceptor count. H_donor: hydrogen bond donor count. B_rotN: rotatable bond count. LogP: oil- water partition coefficient. **(B)** Growth of *E. coli* ATCC 25922 after exposure to different concentrations of 0390 for 18 h. The experiment was repeated three times.

To identify the antibacterial effects, 5,033 unique MINI Scaffold molecules, the molecules in MINI Scaffold Library (TopScience, L5600), were used to combat against standard strains *E. coli* ATCC 25922. The log-phase bacteria were suspended in MH broth to 1 × 10^6^ colony-forming units per milliliter (CFU/ml). To achieve the final concentration of 100 μM, 99 μl of bacteria suspension and 1 μl of tested compounds (the storage concentration is 10 mM) were transferred to 96-well plate. After incubation at 37°C for 16–18 h, the plates were determined the optical density of 630 nm (OD_630_) with a microplate spectrophotometer (Bio-Rad iMark, United States). Finally, only 1 hit (0390, purchased from TopScience) was selected for further study (Figure 1A).

### Antimicrobial susceptibility

The *in vitro* antibacterial effects of drugs were evaluated by a standard microdilution broth sensitivity assay according to the guidelines of the Clinical and Laboratory Standard Institute (CLSI, 2021). In brief, equal volume of serially diluted drug solution and the bacteria suspension containing 1 × 10^6^ CFU/ml was mixed in 96-well plate. After incubation at 37°C for 16–18 h, the OD_630_ results were detected. MIC was the lowest concentration which cannot be observed visible bacterial growth.

### Scanning electron microscopy and transmission electron microscopy

Log phase bacteria were washed and resuspended in fresh LB broth to 3 × 10^8^ CFU/ml, which were treated with 0390 at a concentration of 32 μg/ml (1 × MIC) or 0.1% DMSO at 37°C and 180 rpm for 1 h. The bacteria were collected by centrifuging at 4,000 × g for 8 min and then fixed with 1.5 ml of 2.5% glutaraldehyde at 4°C for another 48 h. The fixed bacteria were washed with 0.1 M cacodylate buffer to remove excess fixative and secondarily fixed with 1% osmium tetroxide in cacodylate buffer for 2 h. The bacteria were washed with cacodylate buffer, dehydrated by successive soakings in 30, 50, 70, 90, and 100% (v/v) ethanol. The SEM images of the samples were obtained by SEM (HITACHI, Tokyo, Japan; Tan et al., 2017).

For TEM observation, the bacteria were treated as described above. And the fixed bacteria were secondarily fixed with 2% osmium tetroxide. After washing with maleate buffer and water, the cells were soaked in maleate buffer containing uranyl acetate for 1 h, and then dehydrated. The samples were sputter-coated with a layer of gold and observed by TEM (HITACHI, Tokyo, Japan; Tan et al., 2017). The aberrant cells were manually and qualitatively distinguished from the normal cells in each image. The percentage of aberrant cells was calculated by ImageJ software.

### Drug combination assay

The combinational antimicrobial effects between 0390 and antibiotics were evaluated through drug combination assays. Equal volume of serially diluted 0390 solution and antibiotics were mixed in 96-well plate in the presence of 1 × 10^6^ CFU/ml bacteria. The OD_630_ was detected after plates were incubated at 37°C for 16–18 h. FICI, the fractional inhibitory concentration index, is a calculated index employed to evaluate the interaction of drugs combination. Based on the formula of FICI, this index can reflect the MIC changes of drugs before and after drugs combination. FICI was calculated with the formula:


$$\;FICI=\frac{MIC_{A}\;\left(combination\right)}{MIC_{A}\;\left(alone\right)}+\frac{MIC_{B}\;\left(combination\right)}{MIC_{B}\;\left(alone\right)}\;$$

FICI ≤ 0.5 indicated synergy, 0.5 < FICI ≤ 4 indicated no interaction, and FICI > 4 indicated antagonism (Wang et al., 2021).

### Growth inhibition assay

Log phase cells were diluted with MH broth in the presence of 0390 and SPR741 or in combination to a final concentration of 1 × 10^6^ CFU/ml, and the bacteria were treated with 0.1% DMSO as a control. The samples were incubated at 180 rpm 37°C, and the OD_630_ value as well as the viability counts were determined at time points 0, 2, 4, 8, 12, and 24 h, respectively. The viable colonies were quantified by CFU counting.

### Hemolysis assay

Healthy human red blood cells (RBC) were purchased from Hemo Pharmaceutical & Biological Co (Shanghai, China). The cells were washed with 1 × PBS and centrifuged at 1,000 × *g* for 5 min. The cells were then resuspended with 1 × PBS to a concentration of 10% (v/v). Equal volume of RBC suspension and indicated concentrations of 0390 were mixed. 0.1% DMSO and 0.1% Triton X-100 were separately used as negative and positive controls. After static incubation at 37°C for 1 h, 100 μl supernatant was removed to a microplate, and the OD_570_ was detected. The hemolysis rate was calculated with the formula (Tan et al., 2019):


$$Hemolysis\;\left(\%\right)=\frac{A_{sample}-A_{0.1\%DMSO}}{A_{0.1\%Triton\;X-100}-A_{0.1\%DMSO}}\times 100\%$$

### Cell viability assay by CCK-8

CCK-8, cell counting kit-8, was used to evaluate cell viability, referring to cell proliferation and cytotoxicity. Cell viability assay using CCK-8 (Tongren, Japan) was carried out to detect the cytotoxicity of 0390, and four different types of cell lines were used to investigate. Human hepatocarcinoma cell line HepG2 was cultured in DMEM supplemented with 10% FBS and 1% Abs, human normal hepatocyte cell line LO2 in PRMI-1640 medium with 10% FBS and 1% Abs, human kidney tubule epithelial cell line HK-2 in MEM with 10% FBS and 1% Abs, and human skin fibroblast cell line HSF were cultured in special culture medium (iCell Bioscience Inc., Shanghai, iCell-0051a-001b) with 10% FBS and 1% Abs. Cells were incubated at 37°C in a humidified atmosphere of 5% CO_2_. One hundred microliters of log-phase cells were transferred to 96-well plates with a density of 5 × 10^3^ cells per well. The adherent cells were treated with indicated concentrations of 0390 for 24 h, and then, respectively, added 0.1% DMSO and CERI to work as negative and positive control. Ten microliters of CCK-8 reagent were plated to each well. After incubation for another 4 h, A450 value was detected.

### Calcein acetoxymethylester/propidium iodide staining assay

The effect of SPR741 and 0390 on the viability of HK-2 was further evaluated by the calcein-AM/PI double stain Kit (Nanjing, China). The transformation of calcein-AM to calcein contributes to green fluorescence in living cells, and the presence of PI leads to red fluorescence in dead cells. HK-2 cells were plated at 1 × 10^4^ cells/cm^2^ into 12-well plate and adherent cells were incubated with SPR741 (32 μg/ml) and 0390 (64 μg/ml) for 24 h. After gently washing with PBS, cells were labeled with the calcein-AM/PI Kit for 30 min and then the living cells (green cytoplasmic fluorescence) and dead cells (red nucleus fluorescence) were observed by an inverted fluorescence microscope.

### Subcutaneous infection model

All animal procedures were performed according to the requirements of the Ethic Committee of the Third Xiangya Hospital, Central South University (NO: 2021sydw0245). Seven-week-old female ICR mice (Hunan SJA Laboratory Animal Co., Ltd., China) weighing 23–27 g were used to develop the subcutaneous infection model. The fur on the back was removed by shaving and chemical depilatories. Overnight-cultured bacteria were washed with saline and 50 μl of bacteria suspension containing 1 × 10^7^ CFU was injected into the right side of the dorsum. Two hours after incubation, mice were randomly divided into four groups (*n* = 6 mice/group), which were treated with 100 μl of 0.5% DMSO (vehicle), 20 mg/kg of SPR741, and 20 mg/kg of 0390 alone or in combination, respectively. Treatments were directly injected subcutaneously into the infected area. After treatment for 24 h, abscess tissues were measured by a caliper. After tissues were excised and homogenized in saline, viable cells were quantified by CFU counting.

### Histological analysis

Infected skin tissues were collected after treatment for 24 h and fixed in 4% paraformaldehyde fix solution for 24 h. The abscess tissues were processed with a series of ethanol and embedded in paraffin blocks. Tissue sections were stained with Hematoxylin–Eosin (H&E) and Masson’s trichrome staining to visualize pathological changes among the four groups (Khalil and Abunasef, 2015).

### Immunohistochemical staining

To explore the effects of drugs on proinflammatory cytokines production, immunohistochemical staining of TNF-α and IL-6 was performed following the manufacturer’s instructions (Servicebio, Wuhan, China). All of the primary antibodies were from Servicebio, Wuhan, China. And the dilution ratios were 1: 1,000 and 1: 800, respectively. Color was developed using a DAB substrate kit. Samples were counterstained with Hematoxylin and dehydrated. The images of samples were observed under an upright epifluorescence microscope (Nikon E100, Japan).

### *In vivo* toxicity

Seven-week-old female ICR mice weighing 23–27 g were randomly divided into three groups (*n* = 5 mice/group), which were treated with 0.5% DMSO (vehicle), 220 mg/kg of SPR741, and 20 mg/kg of 0390 alone or in combination, respectively. After administration subcutaneously for 24 h, the whole blood and plasma of mice were collected for whole blood analysis and organic function biomarker quantification. The organs, including spleens, kidneys, livers, lungs, and hearts were excised and fixed for 24 h in 4% paraformaldehyde fix solution and then were stained by H&E for histopathological analysis.

### Statistical analyses

Experiments were independently performed at least in triplicate. Data were analyzed using the GraphPad Prism 8.0 software and expressed as mean ± SD. *p* values for two-group comparisons were measured using the Student’s *t*-test, and for multiple comparisons, data were analyzed using one-way ANOVA. *p* < 0.05 was considered statistically significant.

## Results

### Antimicrobial effects of 0390 against *Escherichia coli*

**Figure 2:**
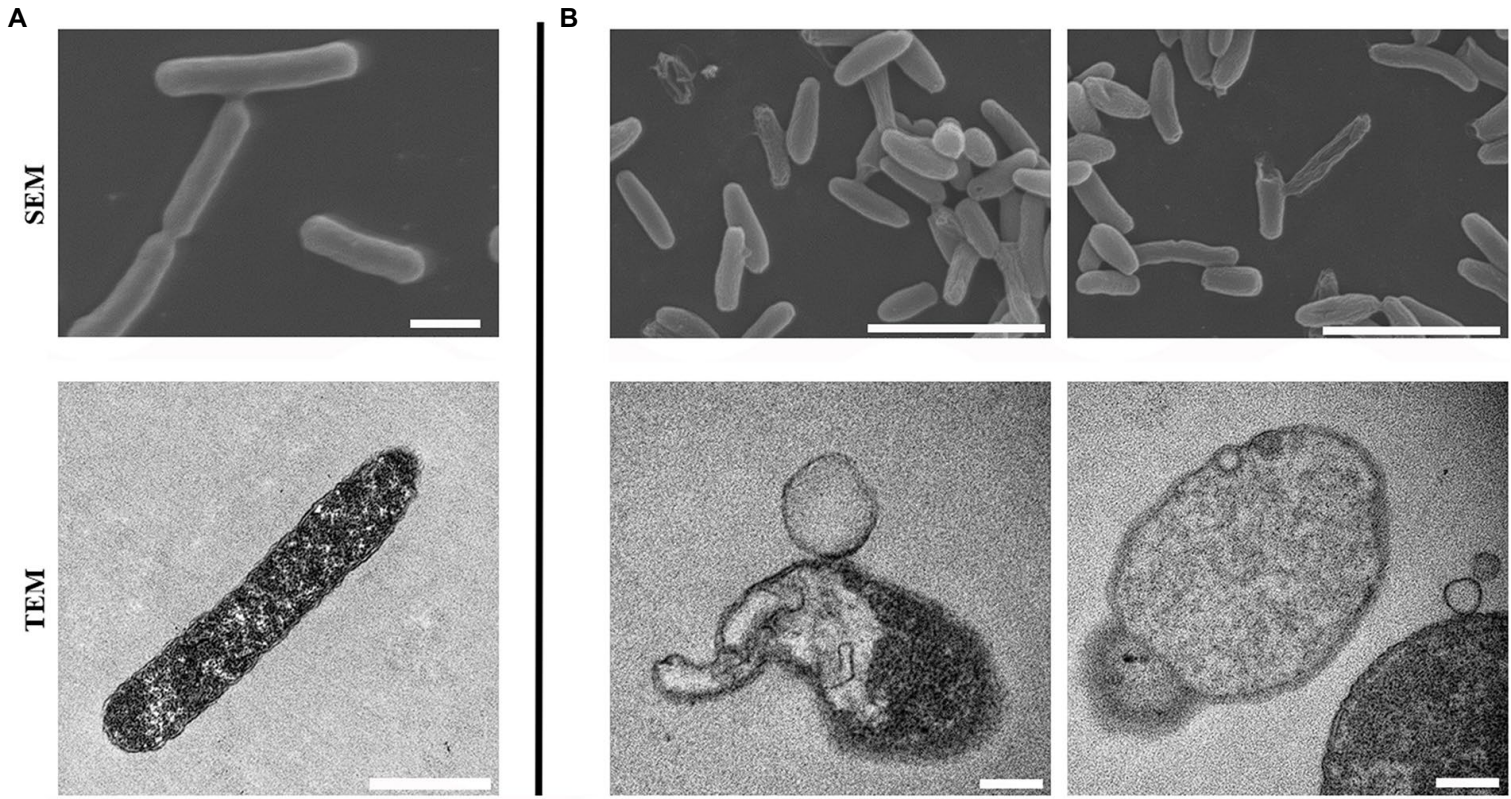
Morphologic and microstructural changes of *Escherichia coli* after being treated with 0390 for 1 h. Scanning electron microscopy (SEM) and transmission electron microscopy (TEM) images of **(A)** untreated, or **(B)** 32 μg/ml of 0390 treated *E. coli* ATCC 25922. Scales for SEM: 5 μm; for TEM: 1 μm.

To develop the potential antibacterial molecules against *E. coli*, we performed high-throughput screening with 5,033 molecules in the MINI Scaffold Library established by TopScience.^1^ High-throughput *in vitro* screening was carried out as described in the method and flowchart of Figure 1A, and only 1 hit from the MINI Scaffold Library had antimicrobial effects against *E. coli* ATCC 25922 (Supplementary Figure S1). The chemical structure of 0390 was shown in Figure 1A. The dose-dependent growth inhibition effect of 0390 was shown in Figure 1B. Furthermore, we conducted primary antimicrobial susceptibility tests of 0390 to other pathogens, including *P. aeruginosa, A. baumannii, K. pneumoniae, E. faecalis, and S. aureus*. However, 0390 exhibited no antimicrobial activity against other pathogens with MIC >64 μg/ml (Supplementary Figure S2). Thus, an electron microscope was performed to observe the morphology and microstructure of 0390-treated *E. coli*. For SEM, the surfaces of rodlike bacteria were smooth and structure-intact in the control group (Figure 2A). However, the appearance of incomplete structure and wrinkles were observed after treatment with 32 μg/ml of 0390 for 1 h (Figure 2B). Similarly, for TEM observation, cell wall disruption, cell membrane bulging, and spillover of cell contents were shown in the presence of 0390 (32 μg/ml), compared with DMSO-treated *E. coli* (Figures 2A,B; Supplementary Figure S3).

### Synergistic bacteriostatic activity between 0390 and SPR741

**Figure 3:**
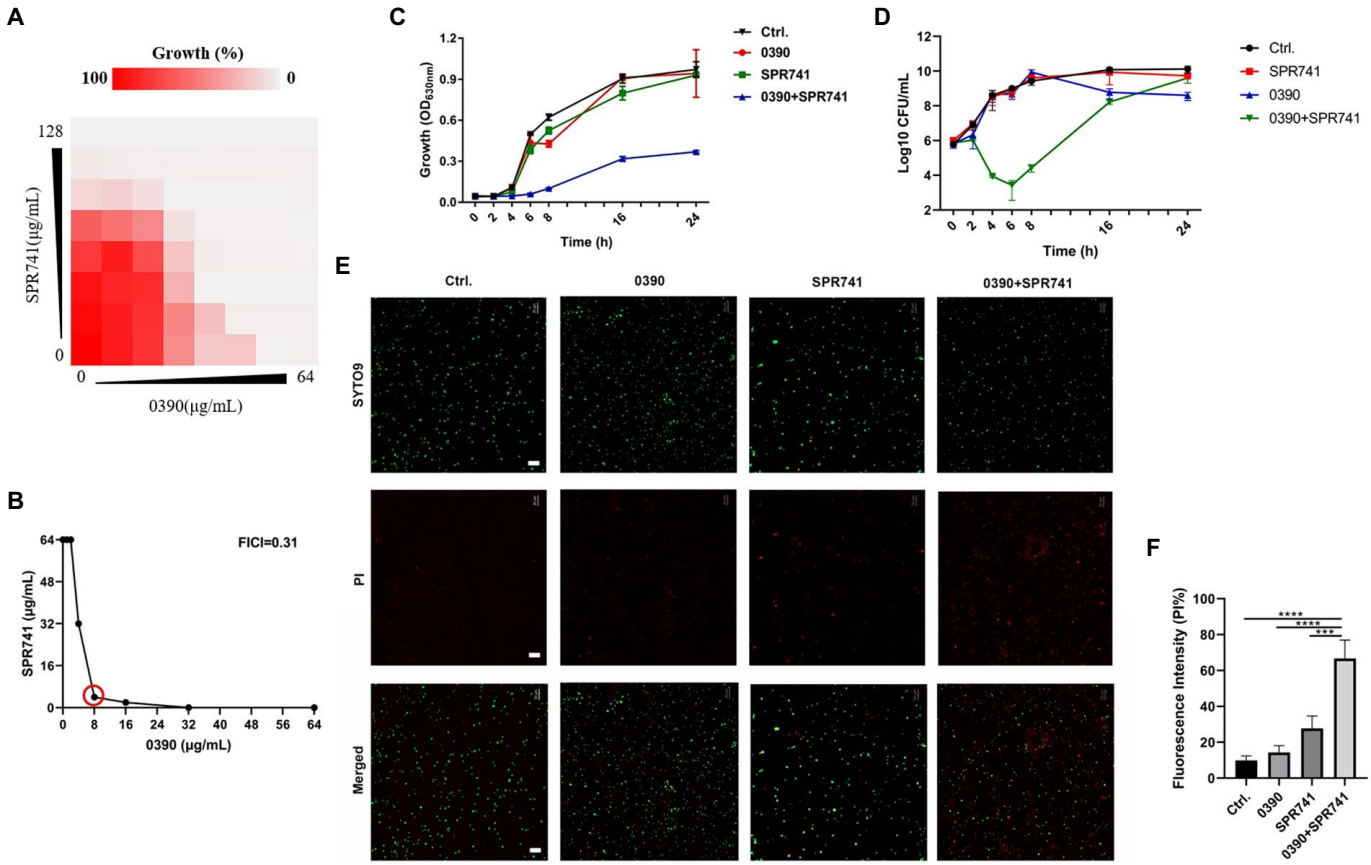
Combinational bacteriostatic effects between 0390 and SPR741 against *Escherichia coli* ATCC 25922. **(A)** Synergistic antimicrobial effects between 0390 and SPR741 by using drug combination assays. **(B)** Calculation of the fractional inhibitory concentration index (FICI). The red circle indicates the optimal FICI. **(C)** The time-growth inhibition curves and **(D)** time-killing curves of *E. coli* in the presence of 0390 (1/4 × MIC) and SPR741 (1/8 × MIC) alone or in combination. The experiment was repeated three times. **(E)** Viable cells visualization detected by SYTO 9 and PI staining, with treatment of 0390 (1/4 × MIC) and SPR741 (1/8 × MIC) alone or in combination. Green indicates live cells and red indicates dead cells. Scale bar: 20 μm. **(F)** Fluorescence intensity analysis of PI-stained cells was carried out by ImageJ software. 100% intensity = the intensity of SYTO9 + the intensity of PI. ^***^p < 0.001, ^****^p < 0.0001.

**Figure 4:**
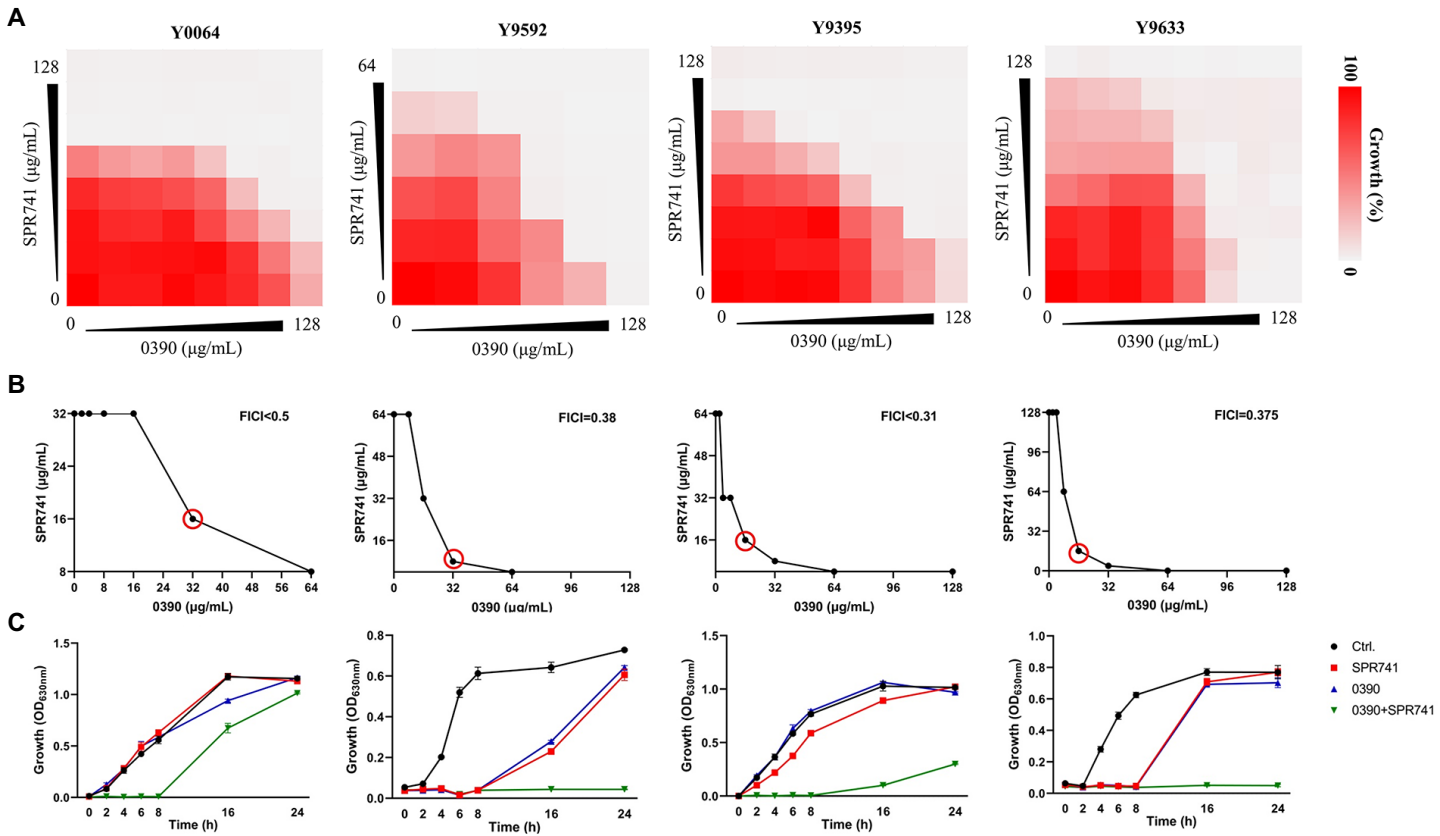
Synergistic antimicrobial activity of 0390 and SPR741 combination against *Escherichia coli* XDR strains. **(A)** Results of the drug combination assays of four *E. coli* XDR strains indicate synergism between 0390 and SPR741. **(B)** Red circles indicate the optimal FICIs. **(C)** Time-growth curves of the XDR strains treated with 0390 and SPR741 alone or in combination (64 μg/ml 0390 + 8 μg/ml SPR741 for Y0064; 16 μg/ml 0390 + 16 μg/ml SPR741 for Y9592; 16 μg/ml 0390 + 16 μg/ml SPR741 for Y9395; and 16 μg/ml 0390 + 16 μg/ml SPR741 for Y9633, respectively). The experiment was repeated three times.

To assess the combinational antimicrobial effects, the interactive activities between 0390 and other drugs against *E. coli* ATCC 25922 were detected by drug combination assays. The FICIs of combination therapies against *E. coli* ATCC 25922 between 0390 and most polypeptide antibiotics, including polymyxin B (PB), polymyxin E (PE), polymyxin B nonapeptide (PMBN), and SPR206, or other conventional antibiotics, such as erythromycin (ERY), gentamicin (GEN), Ceftriaxone sodium (CRO), tetracycline (TET), and as levofloxacin (LEV), indicated no interaction (Supplementary Figure S4). However, significant synergistic effects were observed in the group of 0390 combined with SPR741 with FICI = 0.31 (Figures 3A,B). Besides, the FICIs of the antimicrobial combination between SPR741 and other conventional antibiotics against ATCC 25922 indicated no or only moderate synergistic effects (Table 1). In addition, no interaction or antagonism between 0390 and SPR741 was observed when we changed the objective to other strains, such as *K. pneumoniae, P. aeruginosa, A. baumannii, S. aureus, and S. epidermidis* (Supplementary Figure S3). Thus, the combinational activity between SPR741 and 0390 was species-dependent. In order to verify the synergy between 0390 and SPR741 against *E. coli*, the growth inhibition assays and Live/Dead staining were performed. As shown in Figure 3C, the combination of sub-MIC of SPR741 (8 μg/ml) and 0390 (8 μg/ml) effectively inhibited the bacterial growth within 24 h. Moreover, by CFU counting, a reduction of more than 2 log10 CFU/ml was observed in the group of 0390 and SPR741 in combination, after 6 h treatment compared to the initial bacteria load (Figure 3D). Consistently, SYTO9 and PI staining by CLSM observation revealed that the combined treatment of 0390 and SPR741 destroyed the bacterial viability and resulted in reduced viable cell counts compared with the control or mono-treatment group (Figures 3E,F). Further, we evaluated the synergistic antimicrobial activity of SPR741 and 0390 against drug-resistant clinical isolates of *E. coli*. As shown in Figure 4A, the results of the drug combination assays also indicated the synergistic antimicrobial activity against *E. coli* XDR strains of Y0064, Y9395, Y9522, and Y9633 with FICIs of <0.5, 0.38, <0.31, and 0.375, respectively (Figure 4B). Consistently, the growth inhibition assays suggested that the combination treatment of 0390 and SPR741 possessed a significant synergistic antimicrobial activity compared with single-usage groups (Figure 4C).

**Table 1:** Drug combinations between SPR741 and conventional antibiotics.

Groups	Antibiotics	MIC (μg/ml)		Fold	FICI	Outcome
		Alone	Combined	change		
1	AMP	8	2	0.25	0.5	Synergy
	SPR741	32	8	0.25		
2	GEN	16	8	0.5	0.75	No interaction
	SPR741	32	8	0.25		
3	LEV	0.016	0.008	0.5	1	No interaction
	SPR741	32	16	0.5		
4	IMP	32	4	0.125	0.625	No interaction
	SPR741	32	16	0.5		
5	PB	4	2	0.5	0.5625	No interaction

AMP, ampicillin; GEN, gentamycin; LEV, levofloxacin; IMP, imipenem; and PB, polymyxin B.

### Cytotoxicity assessment of 0390 alone or in combination with SPR741

**Figure 5:**
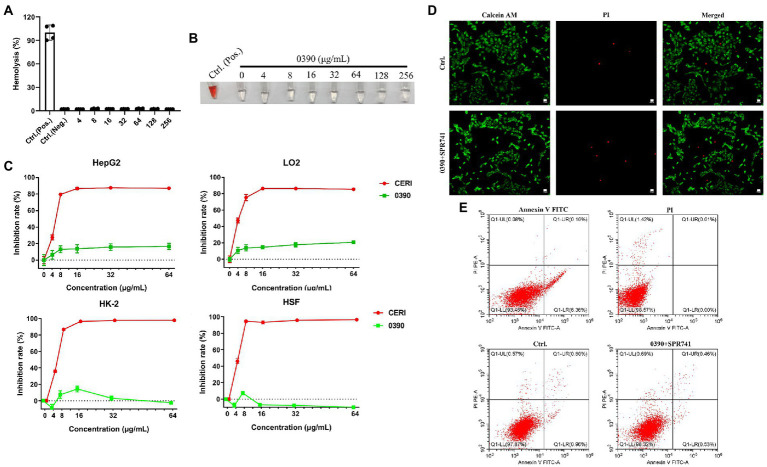
Hemolytic activity and cytotoxicity of 0390 alone or in combination with SPR741. Human RBCs hemolysis rates **(A)** and representative images **(B)** in the presence of a series concentrations of 0390, 0.1% DMSO (negative control), or 0.1% Triton X-100 (positive control). **(C)** The cytotoxicity of 0390 and CERI (positive control) to HepG2 (human hepatocarcinoma cell line), LO2 (human normal hepatocyte cell line), HK-2 (human kidney tubule epithelial cell line), and HSF (human skin fibroblast cell line) were evaluated by CCK-8 assays. **(D)** CLSM images of HK-2 cells stained by calcein-AM/PI after being treated with 0390 alone or in combination with SPR741. Green indicates live cells and red indicates dead cells. Scale: 100 μm. **(E)** HK-2 cells apoptosis analysis by flow cytometry, with stained by Annexin V FITC/ PI. Flow cytometry analyzed the percentages of normal (Q1-LL), early apoptotic (Q1-LR), late apoptotic (Q1-UR), and necrotic cells (Q1-LR + Q1-UR), respectively.

Hemolytic toxicity assays showed that no hemolysis against human RBCs was found with 0390, even at the highest concentration, up to 256 μg/ml (Figure 5A). The related image of the hemolysis assays was shown in Figure 5B. Further, we performed CCK-8 assays to assess the cytotoxicity of 0390 on HepG2, LO2, HK-2, and HSF. Compared with CERI treatment (positive control), 0390 almost showed no growth inhibition to all cell lines at a concentration up to 64 μg/ml (Figure 5C). Due to the consideration of potential nephrotoxicity of SPR741 (Vaara, 2019), we further detected the cytotoxicity of 0390 and SPR741 in combination to the renal derived cell line HK-2. Combination treatment of 0390 (32 μg/ml) and SPR741 (64 μg/ml) did not exhibit death-inducing activity to HK-2 cells by Calcei AM/ PI staining, compared to control group (Figure 5D). Similarly, by comparing the percentages of early apoptotic, late apoptotic, and necrotic cells between groups, the flow cytometry analysis indicated that the combination treatment did not induce apoptosis of the HK-2 cells (Figure 5E). To sum up, 0390 in combination with SPR741 displayed extremely low toxicity *in vitro*.

### *In vivo* synergistic antimicrobial efficacy between 0390 and SPR741

**Figure 6:**
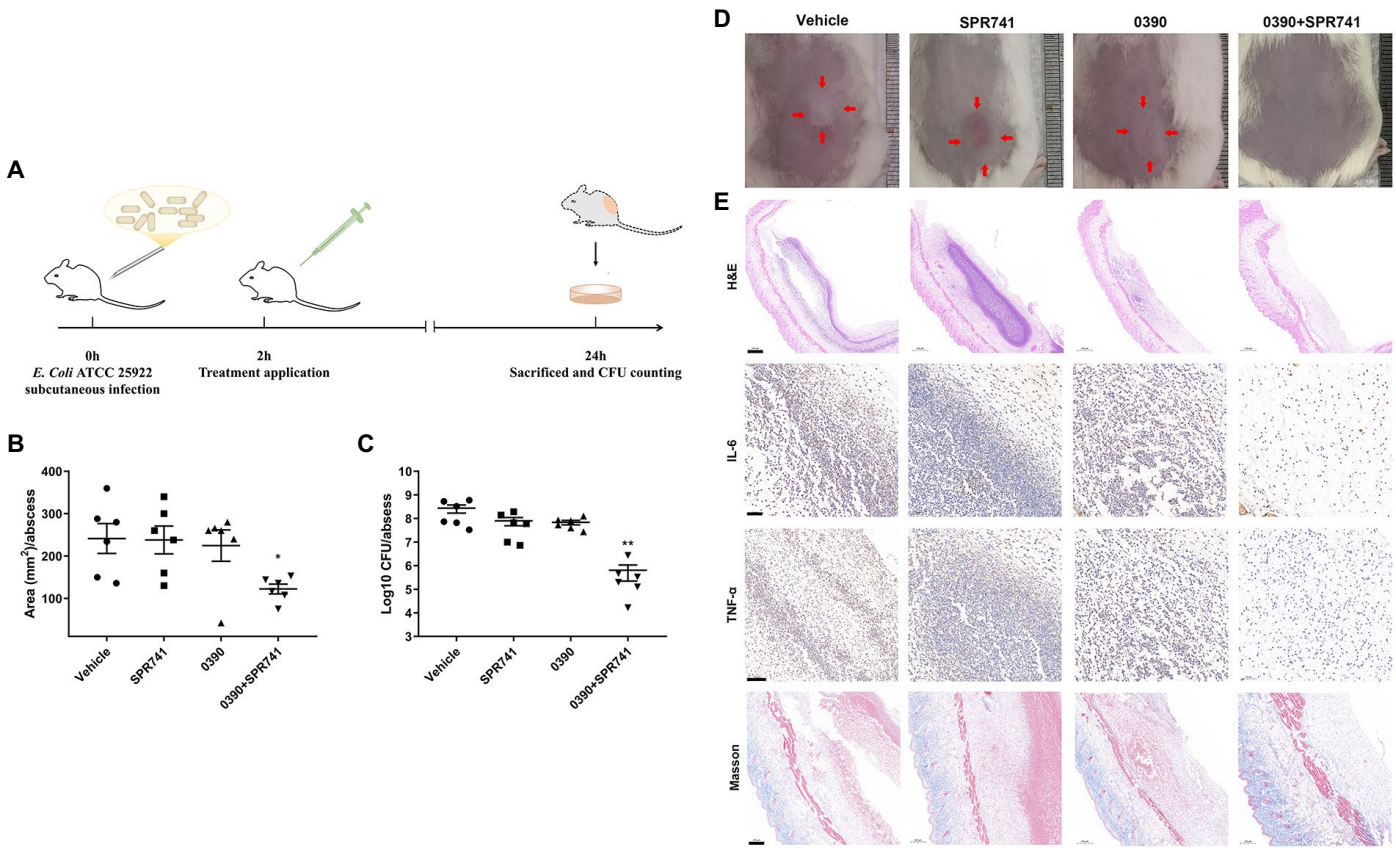
*In vivo* synergistic antimicrobial efficacy of 0390 and SPR741 in combination in a subcutaneous infection model. **(A)** Workflow of the *in vivo* infection model’ construction. Mice (*n* = 6 mice/group) were subcutaneously injected with 0.5% DMSO, 0390, and SPR741 alone or in combination. (Vehicle, 0.5% DMSO; SPR741, 20 mg/kg; 0390, 20 mg/kg; 0390 + SPR741, and 20 + 20 mg/kg). **(B)** Areas, **(C)** viable cell counts, and **(D)** representative images of abscess were detected. **(E)** Abscess histological analysis by using H&E staining, Masson’s trichrome staining, and immunohistochemical staining of IL-6 and TNF-α were evaluated. Vehicle group is used for comparison. ^*^p < 0.05, ^**^p < 0.01.

We performed the subcutaneous abscess models to evaluate the *in vivo* antimicrobial activity, as described in Figure 6A. Even though a mono-therapy with 20 mg/kg of 0390 or SPR741 was not found to be effective against *E. coli* as compared with the vehicle group, the combination treatment showed a significant reduction of the abscess area (Figure 6B), which can be confirmed by visual observation (Figure 6D). As we expected, 0390 and SPR741 in combination also reduced the viable bacteria counts by 2.63 Log10 CFU/mL compared with the vehicle group (Figure 6C). In addition, histological analysis, including H&E staining, Masson’s trichrome staining, and immunohistochemical staining were used to evaluate the production of pro-inflammatory cytokines (TNF-α and IL-6) as well as inflammatory cells’ infiltration. As shown in Figure 6E, the mono-therapy or vehicle group showed significant granulocyte infiltration and pro-inflammatory cytokines aggregation. However, there were no significant pathological morphological changes observed in the combination treatment group, a large number of healthy epithelial cells instead were observed in the abscess.

### Acceptable tolerance of 0390 and SPR741 combination *in vivo*

**Figure 7:**
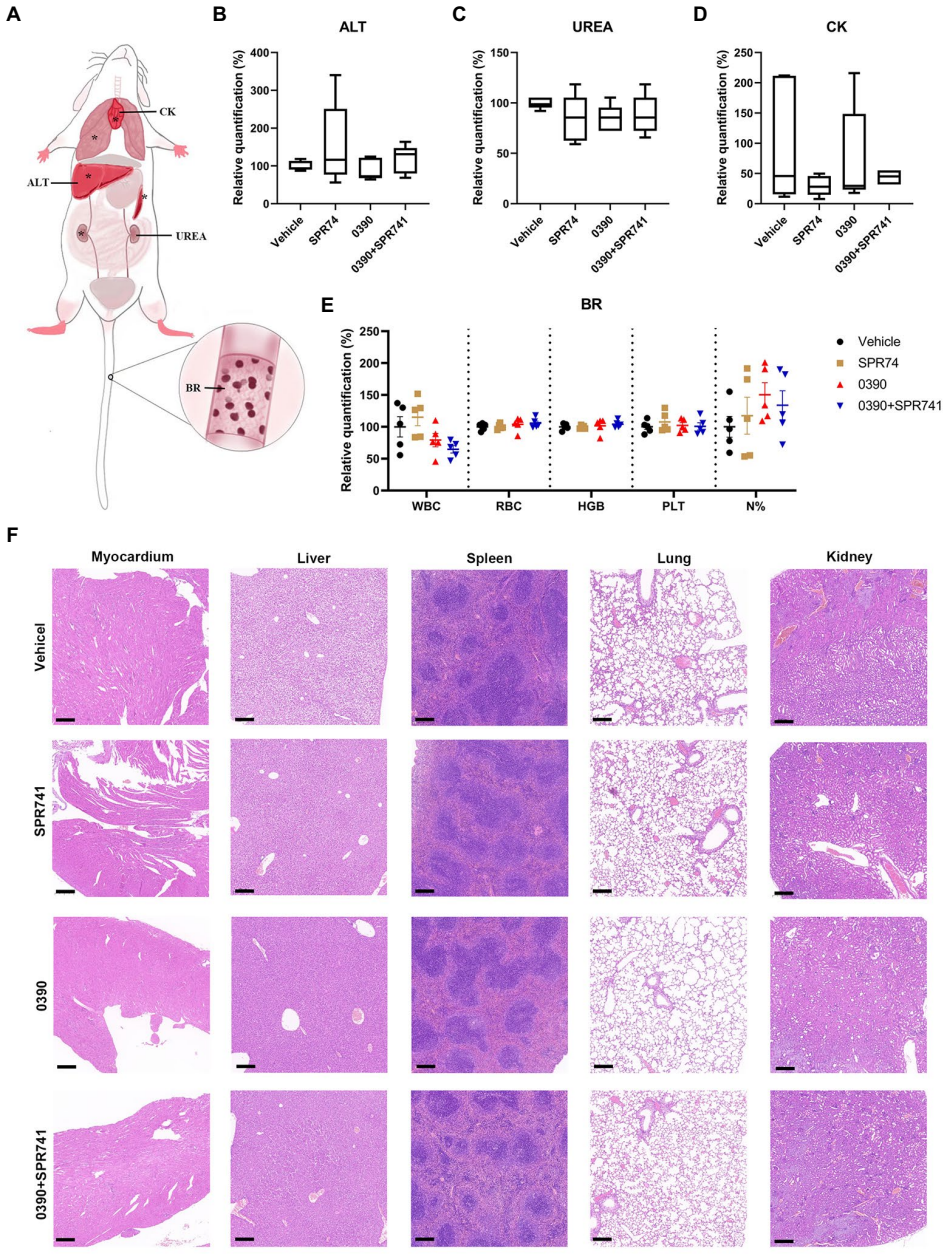
Acceptable *in vivo* tolerance of 0390 and SPR741 in combination. **(A)** Whole body pattern diagram of mice, which is labeled with several biomarkers that reflect corresponding organic functions, blood routine analysis. The organs labeled with ‘*’ were assessed by H&E staining. For *in vivo* toxicity observation, mice (*n* = 5 mice/group) were treated with 0.5% DMSO (Vehicle), 20 mg/kg 0390, and 20 mg/kg SPR741 alone or in combination, respectively. **(B)** Serum alanine transaminase (ALT) quantification for liver function assessment. **(C)** Serum urea (UREA) quantification for renal function assessment. **(D)** Serum creatine kinase (CK) quantification for myocardial function assessment. One-way ANOVA indicted no statistically significant difference among groups. **(E)** Blood routine parameters, including white blood cell counting (WBC), red blood cell counting (RBC), hemoglobin amount (HGB), platelet counting (PLT), and neutrophil classification (N%). One-way ANOVA indicted no statistically significant difference among groups. **(F)** H&E staining of myocardium, liver, spleen, lung, and kidney. Scale: 200 μm.

To investigate the safety profile *in vivo*, several hematological parameters, functional biomarkers quantification, and H&E staining for some crucial organs were assessed by subcutaneous administration of 20 mg/kg of 0390 and SPR741 alone or in combination (Figure 7A). Apparently, there was no statistically significant difference in the biomarkers of ALT (Figure 7B), UREA (Figure 7C), and CK (Figure 7D) among the control, mono-therapy, and combinational therapy groups. Additionally, the hematological biomarkers analysis (including white blood cell counting, red blood cell counting, hemoglobin amount, platelet counting, and neutrophil classification) showed no statistically significant difference among these groups (Figure 7E). Similarly, the images of H&E staining indicated that the treatment of 0390 and SPR741 alone or in combination did not cause histopathological changes in organs including hearts, livers, spleens, lungs, and kidneys compared with the vehicle group (Figure 7F). Accordingly, these findings suggested that the treatment combination demonstrated good safety *in vivo*.

## Discussion

Nowadays, the prevalence of drug-resistant bacteria and lack of novel antibiotics have posed critical issues that result in threats to human health. Antibiotics and bioactive molecules screening are according to specific phenotypic traits, rather than traditional target-based procedures, which can give us ideas that we have never thought of or discovered before. Though it can be a very hard and time-consuming process, several studies have proved satisfactory results in the discovery of antimicrobial compounds with novel mechanisms (Watamoto et al., 2015; Imai et al., 2019). The MINI Scaffold library contains a total of 5,033 molecules with its own scaffold based on more than 16,000,000 drug-like small molecules from ChemDiv. A molecule in the MINI Scaffold Library represents a unique scaffold of a class of compounds, giving rise to the great variety of structures among different molecules, which refers to high diversity. Scaffold is the essence of a molecule and different compounds with the same scaffold generally have higher relevance to the target of action. Thus, discovering a new scaffold can lead us to find a branch of new and effective drugs (Mok and Brenk, 2011). Besides, all molecules in the MINI Scaffold Library were screened and filtered through their pharmaceutical and chemical properties, and adverse structures, such as toxicity and PAINS, were eliminated by TopScience, Shanghai, China. To the best of our knowledge, our study is the first one to report the screening of the MINI Scaffold library against Gram-negative bacterial strains. Due to the existence of outer membrane (OM), Gram-negative bacillus is naturally drug-resistant to many antibiotics, leading to less amount of hits than the Gram-positive bacteria during the screening(Pengfei et al., 2022). However, we found 0390, the only one hit among these molecules with antimicrobial activity specifically against *E. coli*. 0390 is derived from the MINI Scaffold Library, whose molecules are all representative scaffold compounds, and it is very different from antibiotics used in clinical practice. 0390 consists of a triazoloquinazoline core which is substituted with a trifluoroacetamide group on one side and ethyl moiety on the other side, which contributes to antibacterial activities of the compound. Additional studies to assess the role of these groups are warranted.

0390 demonstrated particular antibacterial effects against *E. coli*. The varied susceptibilities of 0390 may be due to the different cell wall components, structure, and genetic backgrounds among species. For example, different from the Gram-positive pathogens, the Gram-negative bacteria had lipopolysaccharide (LPS) structure on the outer membranes as well as cytoplasmic membranes, which could explain their different susceptibilities to polymyxin antibiotics (Falagas and Kasiakou, 2005; Sabnis et al., 2021).

Combination therapy is a hopeful solution to the crisis of antimicrobial resistance. Combination therapies possess several key features, such as greater antibacterial effects than mono-therapy; reduced toxicity companioned with lower doses of drugs in combination; reduced incidence of resistance, etc. (Bollenbach, 2015; Zhou et al., 2015; Kerantzas and Jacobs, 2017; Xu et al., 2018). In the present study, although 0390 exhibits antibacterial activity against *E. coli*, the efficacy of mono-therapy is unsatisfactory with the MIC of 32 μg/ml. Thus, we decide to further investigate the combination therapy between 0390 with other antibiotics. Given that polymyxins like PB, PE, SPR741, and SPR206 can effectively combat multidrug-resistant (MDR) Gram-negative bacillus, and become the only available drugs for MDR Gram-negative bacteria (Phe et al., 2014). However, the toxicity of polymyxins has significantly limited their clinical applications (Akhoundsadegh et al., 2019). Thus, antibiotics in combination with 0390 could be a resort to alleviate the nephrotoxicity of polymyxins.

SPR741, as a derivate of polymyxin B, obtains the ability to disrupt the outer membrane of Gram-negative bacteria with higher safety than polymyxin B (Zurawski et al., 2017). These characters contribute SPR741 to acting as a potential adjutant, in combination with other antimicrobials that can synergistically improve the antimicrobial effects against Gram-negative strains (French et al., 2020). For example, several studies have reported that SPR741 potentiates the activity of conventional antibiotics like fusidic acid, vancomycin, rifampin, minocycline, or beta-lactam against Gram-negative bacteria (Zurawski et al., 2017; Mendes et al., 2020a,b; Alamneh et al., 2022). According to the advantageous features of SPR741, we assume that there are synergistic effects between 0390 and SPR741. In this study, as we expected, we discovered the synergistic antimicrobial activities between 0390 and SPR741 against *E. coli* and its drug-resistant clinical strains. However, 0390 in combination with other classes of drug indicates no interaction. Different classes of antibiotics show antibacterial effects through different model of actions. And the cell wall or membrane disruptors, like SPR741, prone to show synergistic antimicrobial activities with antibiotics targeting inner cellular components. Similarly, Zurawski et al. reported that SPR741 could be synergy with rifampin against XDR *A. baumannii* (Zurawski et al., 2017). Corbett et al. shown that SPR741 demonstrated synergistic activities with azithromycin to combat *E. coli*, *K. pneumoniae*, and *A. baumannii* (Corbett et al., 2017). Thus, based on SPR741 targeting the outer membrane of Gram-negative strains, we assume that is the reason why the synergistic antimicrobial effects are observed. In consistence, there are no synergistic effects observed between the combination of 0390 and other non-outer membrane targeting polymyxins, such as PB, PE, PMBN, and SPR206.

According to the time-killing curve, 0390 in combination with SPR741 demonstrated synergistic bacteriostatic activities against *E. coli* ATCC 25922. However, the bacterial growth is rescued after the time point of 6 h treatment. Other studies also reported similar regrowth during the time-killing curve (Yau et al., 2009; Huang et al., 2017; Alfei et al., 2021). Skarp et al. made hypotheses for the bacteria regrowth phenomenon, such as drug degradation, inoculation effect, and selection for existing or emerging resistant subpopulation (Skarp et al., 2019). But triple combinations could be a resort for sustaining the bacteriostatic activities and theoretically preventing bacteria regrowth (Li et al., 2018; Skarp et al., 2019). Further studies to assess such combinations are needed.

In the present study, our data show that the combination treatment of 0390 and SPR741 indicates antagonism effects against *P. aeruginosa* (Supplementary Figure S3). Antagonism is interpreted as drug interactions which produce an overall activity less than the sum of the two individual drugs, eventually leading to an attenuated activity (Acar, 2000). Drug antagonism is emerging as a powerful tool to study underlying mechanisms of drug action (Yeh et al., 2009). Previously, several studies have shown that, when drug combinations indicate pharmacodynamically antagonistic effects, there are two possible combinational target relationships: one is the two drugs have the same target; the other one is the two drugs have different targets, which affect related pathways and regulate the same phenotype (Jia et al., 2009). Based on this theory, we can conduct further investigations to better understand the action mechanism of 0390.

*In vitro* and *in vivo* toxicity assays all show that 0390 alone or in combination with SPR741 has good safety. According to this, we evaluated the synergistic therapeutic effects between 0390 and SPR741 *in vivo*. Skin and soft tissue infections have been prevalent and increased significantly since the mid-1990s, leading to an increasing clinical and economic burden on health facilities and hospitalized patients (Amin et al., 2014). A Report from the SENTRY Antimicrobial Monitoring program claimed that the occurrence rate of *E. coli* isolated from skin and soft tissue infections is 6.9% (Moet et al., 2007). Besides, to minimize the differences derived from the pharmacokinetics of different species, we constructed subcutaneous abscess infection murine model. As we expected, synergistic antimicrobial activities were observed by the combinational treatment of 0390 and SPR741 *in vivo*. However, we cannot deny that 0390 obtains a very low solubility in water even at a relatively low concentration (no more than 20 mg/kg), which may affect its antimicrobial effects to a certain extent. The water solubility enhancement is necessary and can be optimized by molecule-modified, nano-technology, and complexation with other compounds such as large ring cyclodextrins and protein hydrolysate (Inada et al., 2013; Da Silva et al., 2020; Ismail et al., 2021).

In conclusion, we identified a novel bioactive molecule, 0390, which shows antibacterial activity against *E. coli*. Besides, 0390 combined with SPR741 exhibited significant synergistic combinational effects against both standard strains and drug-resistant clinical strains. In addition, the combination of 0390 and SPR741 significantly decreases the viable bacterial loads in the *E. coli*-induced subcutaneous abscess model *in vivo*. Our results presented here are encouraging due to the bright future of SPR741 in combination with 0390, which could be a promising treatment in the fight against *E. coli* and its drug-resistant strains-related infections, but the underlined mechanism still needs to be investigated. The action mechanism of SPR741 is solely destroying the outer membrane structure, often potentiating the penetration of co-administered antibiotics to exhibit synergistic antimicrobial effects against Gram-negative bacteria. Thus, we hypothesize that 0390 could target inside components of the bacterial cells, like protein synthesis, DNA replication, ROS production, and so on. And further investigations to validate our hypothesis about the mechanisms will be studied in the future.

## Data availability statement

The original contributions presented in the study are included in the article/Supplementary material, further inquiries can be directed to the corresponding author.

## Ethics statement

The animal study was reviewed and approved by the Ethic Committee of the Third Xiangya Hospital, Central South University (NO: 2021sydw0245).

## Author contributions

SP and WY designed and perfected the research. LL conducted most of the experiments and wrote this manuscript. SP, LS, LY, and LZ analyzed research results. LL, YY, and ZL conducted the experiments and data collection. SP and WY assisted with revision and editing the manuscript. All authors contributed to the article and approved the submitted version.

## Funding

This study was supported by the National Natural Science Foundation of China, grant numbers 82072350 and 82202591, the Natural Science Foundation of Hunan Province, grant numbers 2021JJ40944 and 2022JJ70046, and the Key Research and Development Program of Hunan Province of China, grant number 2022SK2116.

## Conflict of interest

The authors declare that the research was conducted in the absence of any commercial or financial relationships that could be construed as a potential conflict of interest.

## Publisher’s note

All claims expressed in this article are solely those of the authors and do not necessarily represent those of their affiliated organizations, or those of the publisher, the editors and the reviewers. Any product that may be evaluated in this article, or claim that may be made by its manufacturer, is not guaranteed or endorsed by the publisher.

## Footnotes

^1^ https://www.tsbiochem.com/
