# Supplementary material for: Identification of a small molecule 0390 as a potent antimicrobial agent to combat antibiotic-resistant Escherichia coli
Source: Front Microbiol. 2022 Dec 15;13:1078318. doi: 10.3389/fmicb.2022.1078318 (PMC9800007; doi:10.3389/fmicb.2022.1078318)
Supplement: Supplementary file 1 [file Data_Sheet_1.zip › 107318 Supplementary_Material 2.docx]

Supplementary Material

# Supplementary methods

## Confocal laser scanning microscopy (CLSM)

Log phase bacteria were washed and suspended in 1xPBS (pH=7.4) to OD_630_ = 0.1 in the presence of 8 μg/mL SPR741 alone or in combination with 8 μg/mL 0390, and 0.1% DMSO was used as a control to eliminate the effects of solvent. After incubation at 37℃ for 2h, the bacteria were collected and resuspended in 1 mL 1xPBS. The samples were added with 10 μM of SYTO9 and Propidium Iodide (PI) (Thermo Fisher Scientific, Shanghai, China). After incubating in the dark for 15 min, the excess SYTO9 and PI were washed out, and the bacteria were resuspended in 1×PBS. The CLSM images were visualized by CLSM (Zeiss LSM800, Jena, Germany), with excitation and emission wavelengths of 485 nm/530 nm and 485 nm/630 nm for SYTO9 and PI, respectively[1]. The fluorescence intensity of the whole image was measured by ImageJ software. 100% intensity = the intensity of SYTO9 + the intensity of PI.

## Cell apoptosis determination by flow cytometry

The cell apoptosis analysis was performed using an Annexin V-fluorescein isothiocyanate (FITC)/PI apoptosis kit (Nanjing, China). Adherent HK-2 cells were incubated with SPR741 (32μg/mL) and 0390 (64μg/mL) for 24 h and digested by EDTA-free pancreatin. The cells were suspended with 500 μL binding buffer containing 5 μL Annexin V and 5 μL PI, incubating for 10 min in the dark, and samples were measured by a flow cytometer in 1 h.

**Reference:**

1. Liu, T., Liu, Y., Liu, M., Wang, Y., He, W., Shi, G., et al., *Synthesis of graphene oxide-quaternary ammonium nanocomposite with synergistic antibacterial activity to promote infected wound healing.* Burns Trauma, (2018) **6**: p. 16 DOI: 10.1186/s41038-018-0115-2.

# Supplementary figures


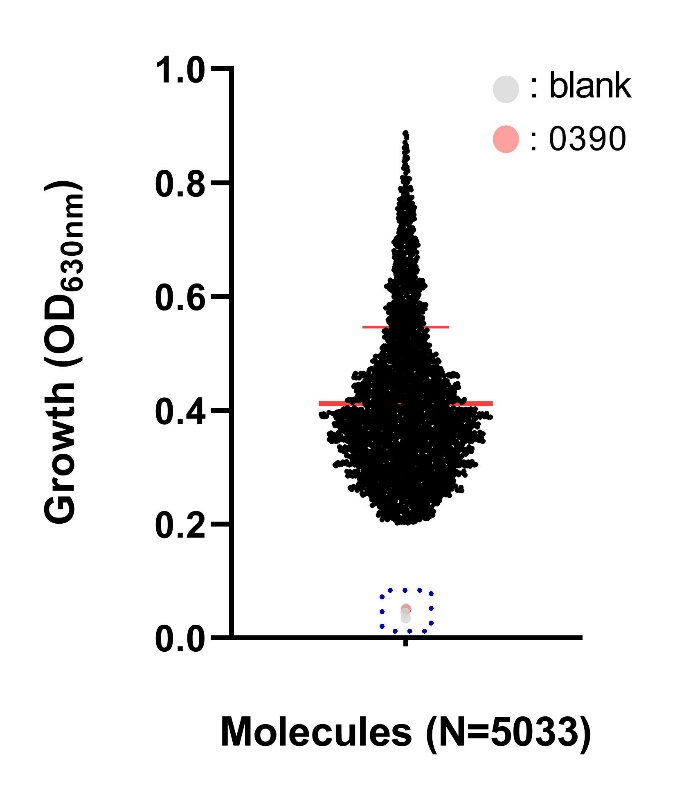


**Figure S1** Identification of antimicrobials against *E. coli* ATCC 25922 from the MINI Scaffold Library using high-throughput screening assays.


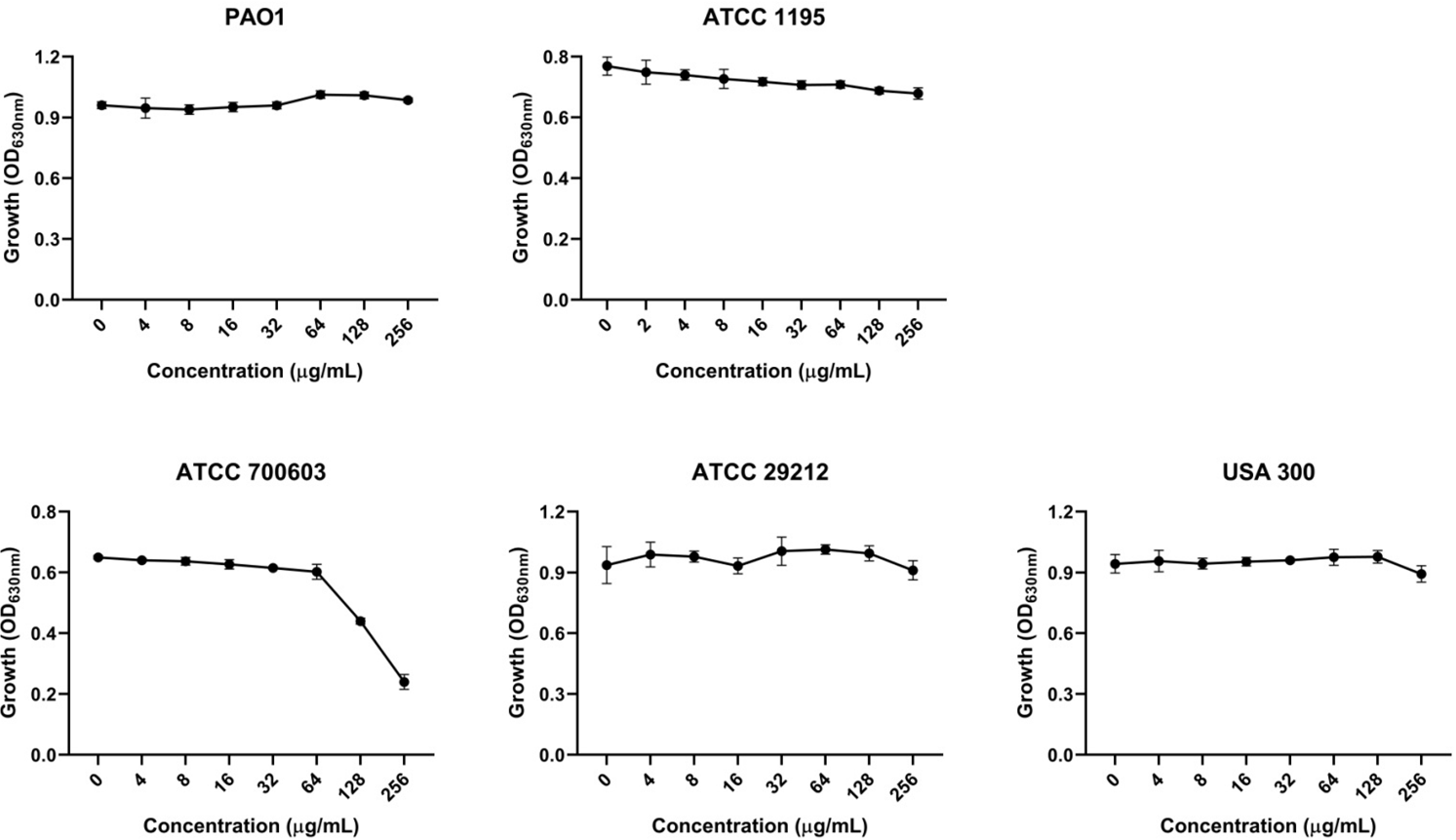


**Figure S2** Antimicrobial sensitivity tests of 0390 by microplate dilution method against type strains of *P. aeruginosa* PAO1, *A. baumannii* ATCC 1195, *K. pneumoniae* ATCC700603, *E. faecalis* ATCC 29212, and *S. aureus* USA 300. The experiment was repeated three times.


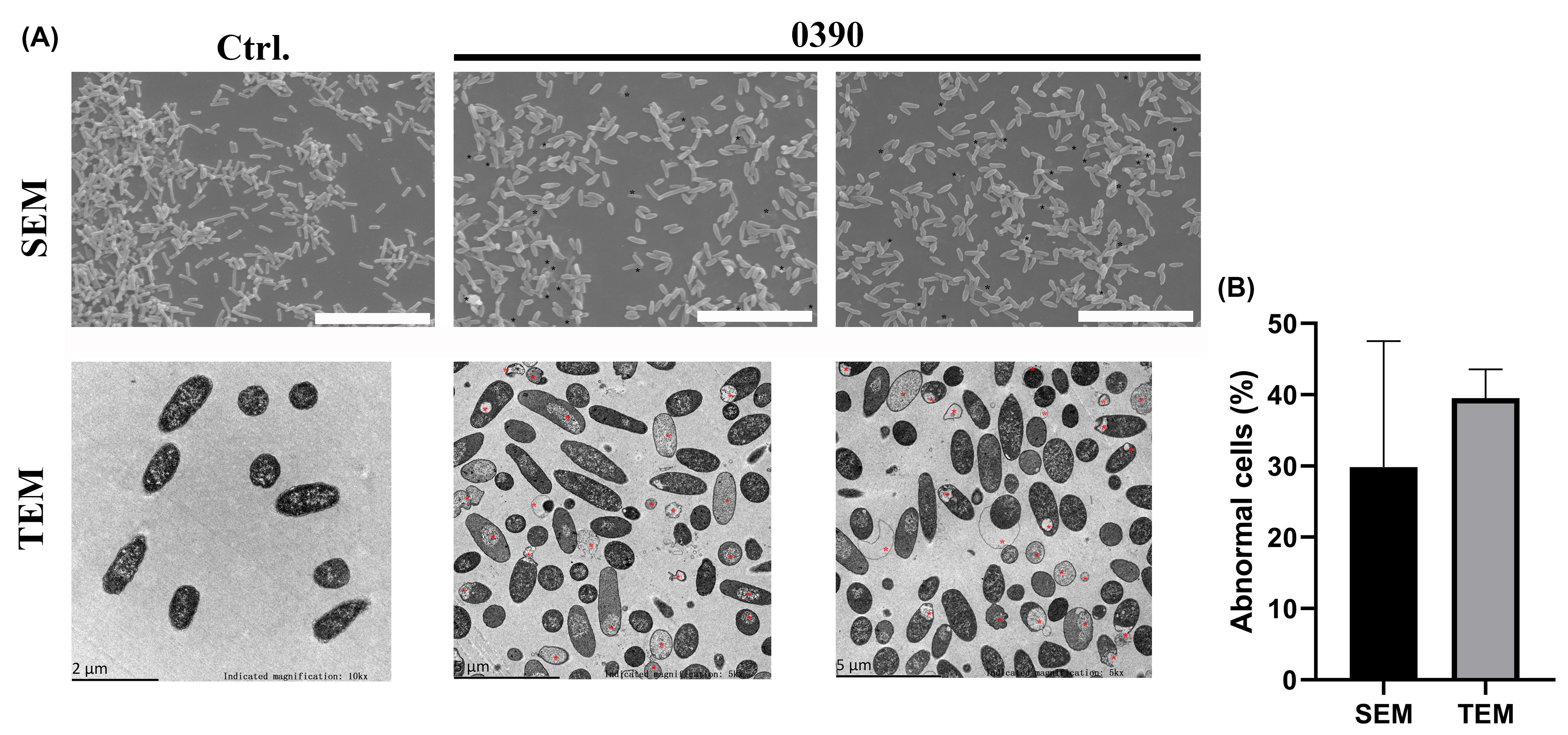


**Figure S3** (A) Scanning electron microscopy (SEM) and transmission electron microscopy (TEM) images of untreated, or 0390 (32 μg/mL) treated *E. coli* ATCC 25922. The black asterisks in SEM indicate the aberrant cells; The red asterisks in TEM indicate the aberrant cells. Scale for SEM: 20 μm. (B) The percentages of abnormal *E. coli* ATCC 25922 cells in SEM and TEM images after being treated with 32 μg/mL of 0390, analyzed by ImageJ software. Aberrant cells were defined as cells with abnormal structures compared with the control, such as cell enlargement, atrophy, vacuolation, deficiency and depression, et al. The error bars indicate the standard deviation.


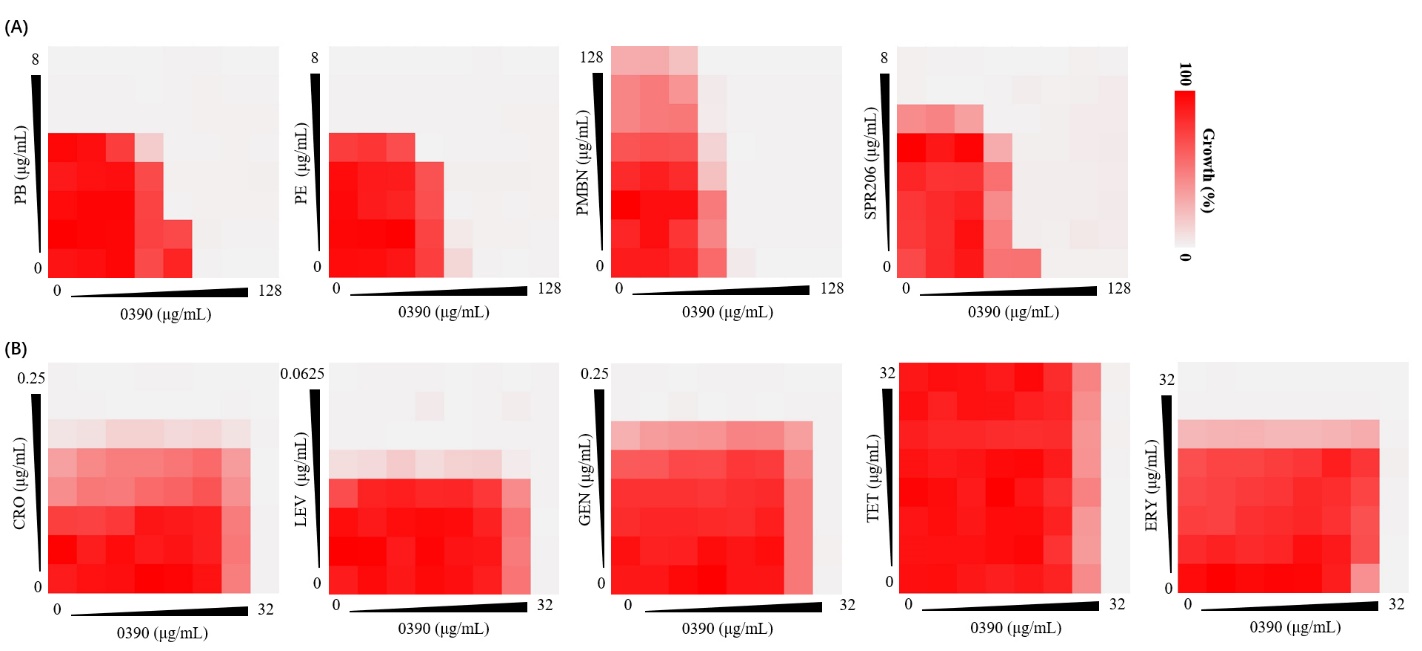


**Figure S4** Drug combinations between 0390 and polypeptide antibiotics (A) or other conventional antibiotics (B) against *E. coli* ATCC 25922. PB, polymyxin B; PE, polymyxin E; PMBN, polymyxin B nonapeptide; ERY, erythromycin; GEN, gentamicin; CRO, Ceftriaxone sodium; TET, tetracycline; LEV, levofloxacin. The experiment was repeated three times.


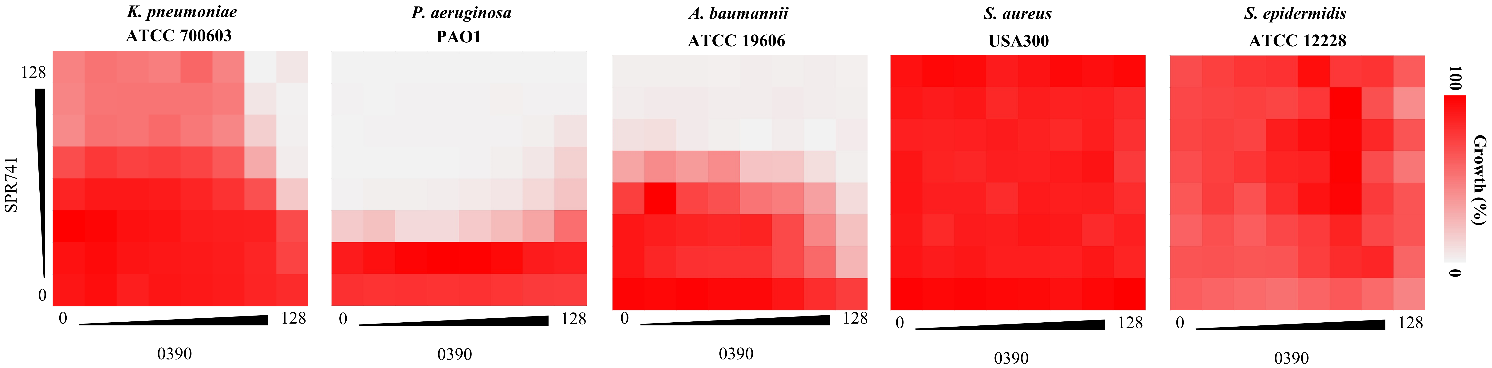


**Figure S5** Drug combination between 0390 and SPR741 against common pathogens, including *K. pneumoniae* ATCC700603, *P. aeruginosa* PAO1, *A. baumannii* ATCC 19606, *S. aureus* USA 300, and *S. epidermidis* ATCC 12228. The experiment was repeated three times.

# Supplementary table

**Table S1** Strains used in this study

| Strains | Resistant pattern | Source |
| --- | --- | --- |
| Gram-negative | | |
| *E. coli* |  |  |
| ATCC 25922 | Standard strain, Non-MDR | ATCC |
| Y0064 | XDR | Clinical isolated |
| Y9395 | XDR | Clinical isolated |
| Y9592 | XDR | Clinical isolated |
| Y9633 | XDR | Clinical isolated |
| *P. aeruginosa* |  |  |
| PAO1 (ATCC 15692) | Standard strain, Non-MDR | ATCC |
| *A. baumanii* |  |  |
| ATCC 1195 | Standard strain, Non-MDR | ATCC |
| ATCC 19606 | Standard strain, Non-MDR | ATCC |
| *K. pneumoniae* |  |  |
| ATCC 700603 | Standard strain, Non-MDR | ATCC |
| Gram-positive | | |
| *S. aureus* |  |  |
| USA300 | Standard strain, MRSA | ATCC |
| *S. epidermidis* |  |  |
| ATCC 12228 | Standard strain, Non-MDR | ATCC |
| *E. faecalis* |  |  |
| ATCC 29212 | Standard strain, Non-MDR | ATCC |

MDR: multidrug-resistant; XDR: extensively drug-resistant.
