# Supplementary figures and images for: Identification of a small molecule 0390 as a potent antimicrobial agent to combat antibiotic-resistant Escherichia coli
Source: Front Microbiol. 2022 Dec 15;13:1078318. doi: 10.3389/fmicb.2022.1078318 (PMC9800007; doi:10.3389/fmicb.2022.1078318)

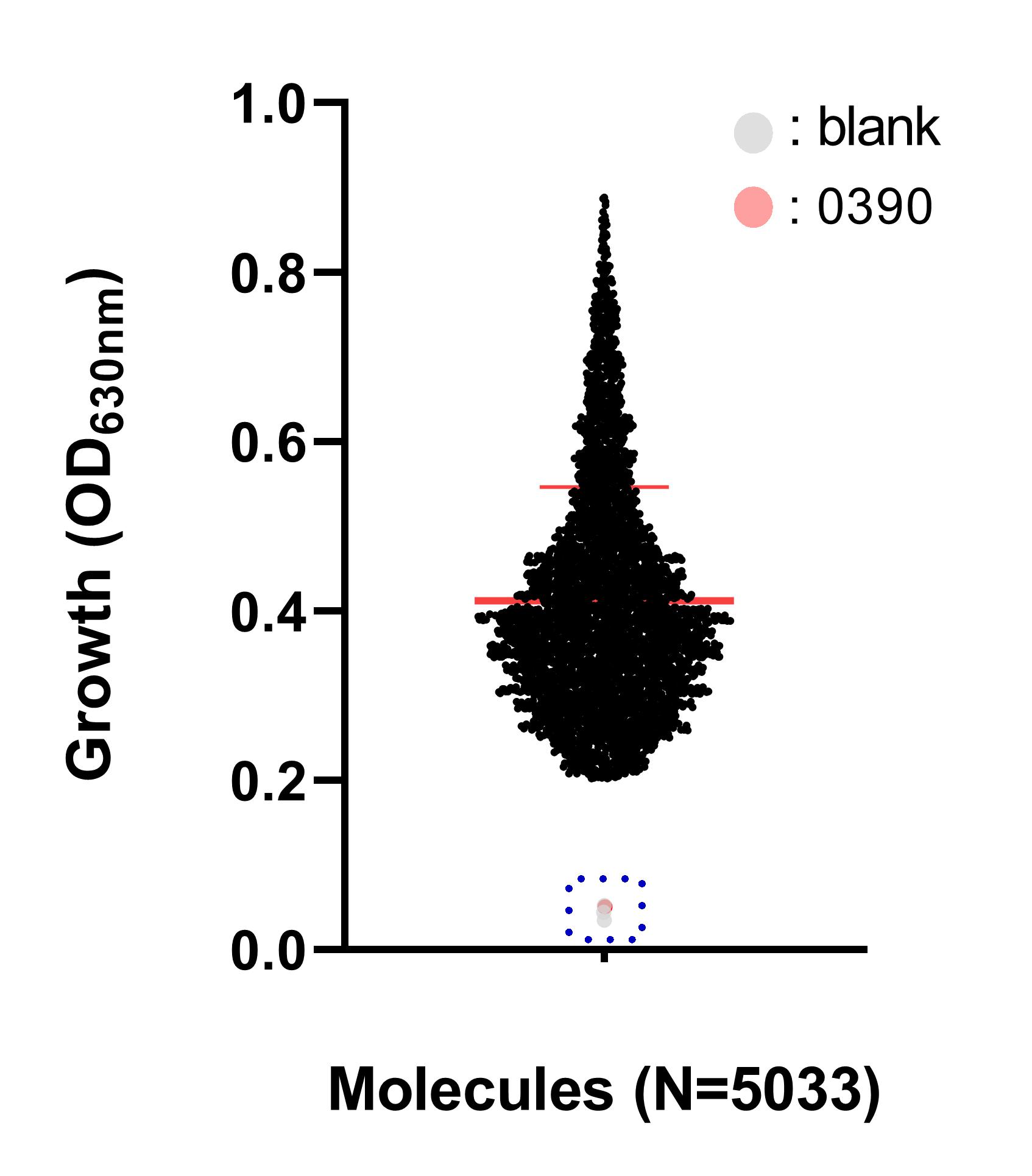

Supplement: Supplementary file 1 [file Data_Sheet_1.zip › Figure S1.jpg]

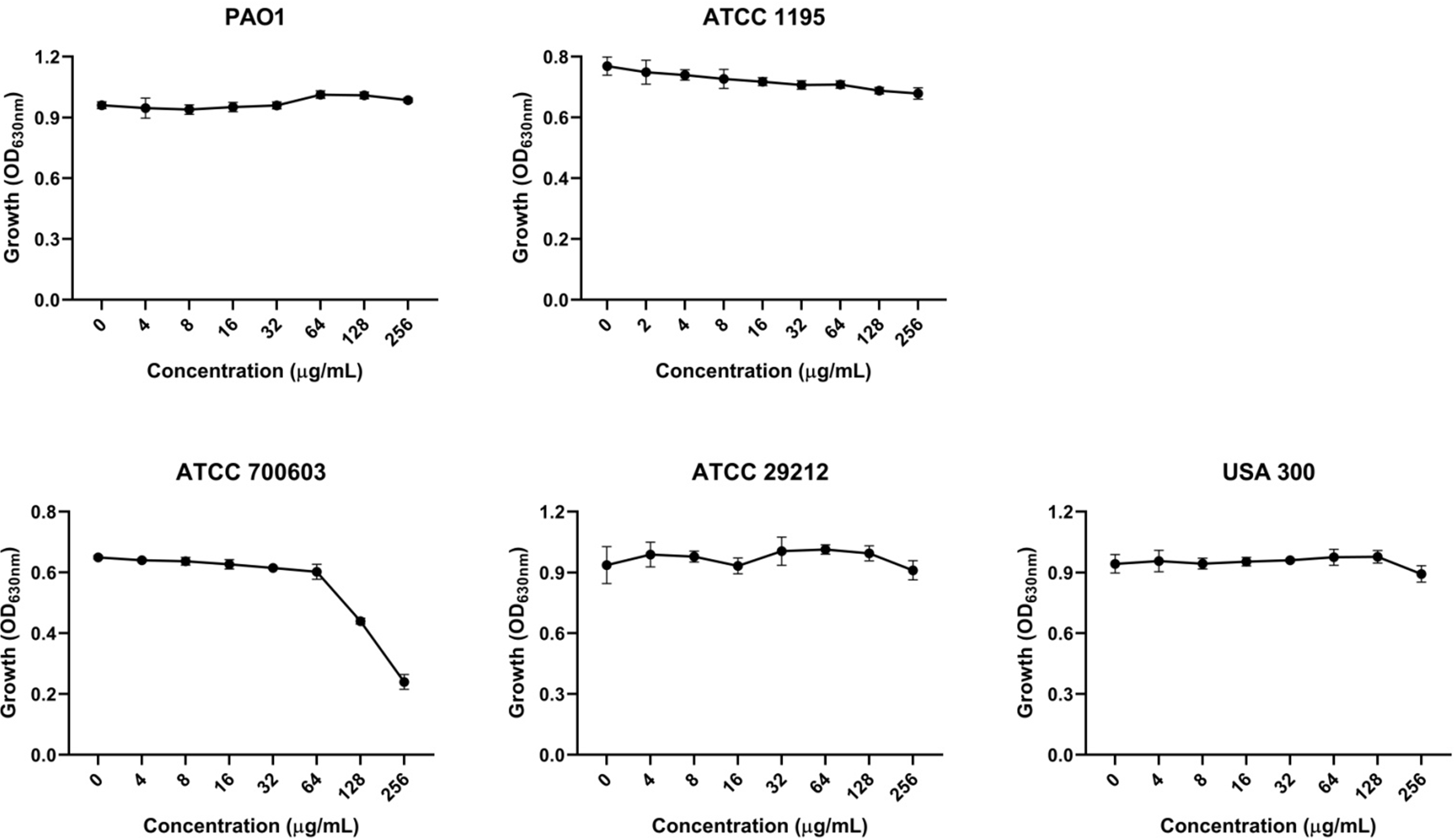

Supplement: Supplementary file 1 [file Data_Sheet_1.zip › Figure S2.tif]

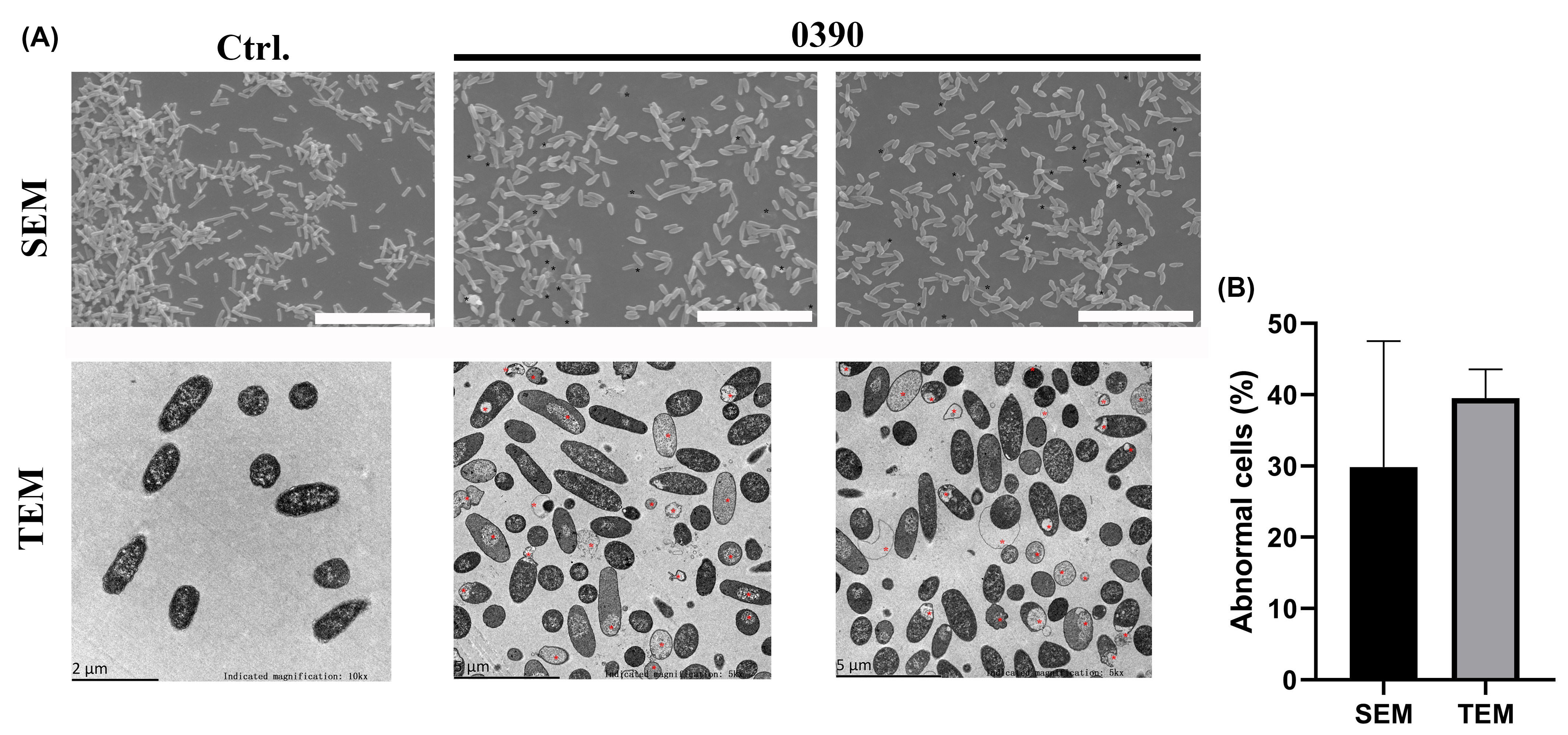

Supplement: Supplementary file 1 [file Data_Sheet_1.zip › Figure S3.jpg]

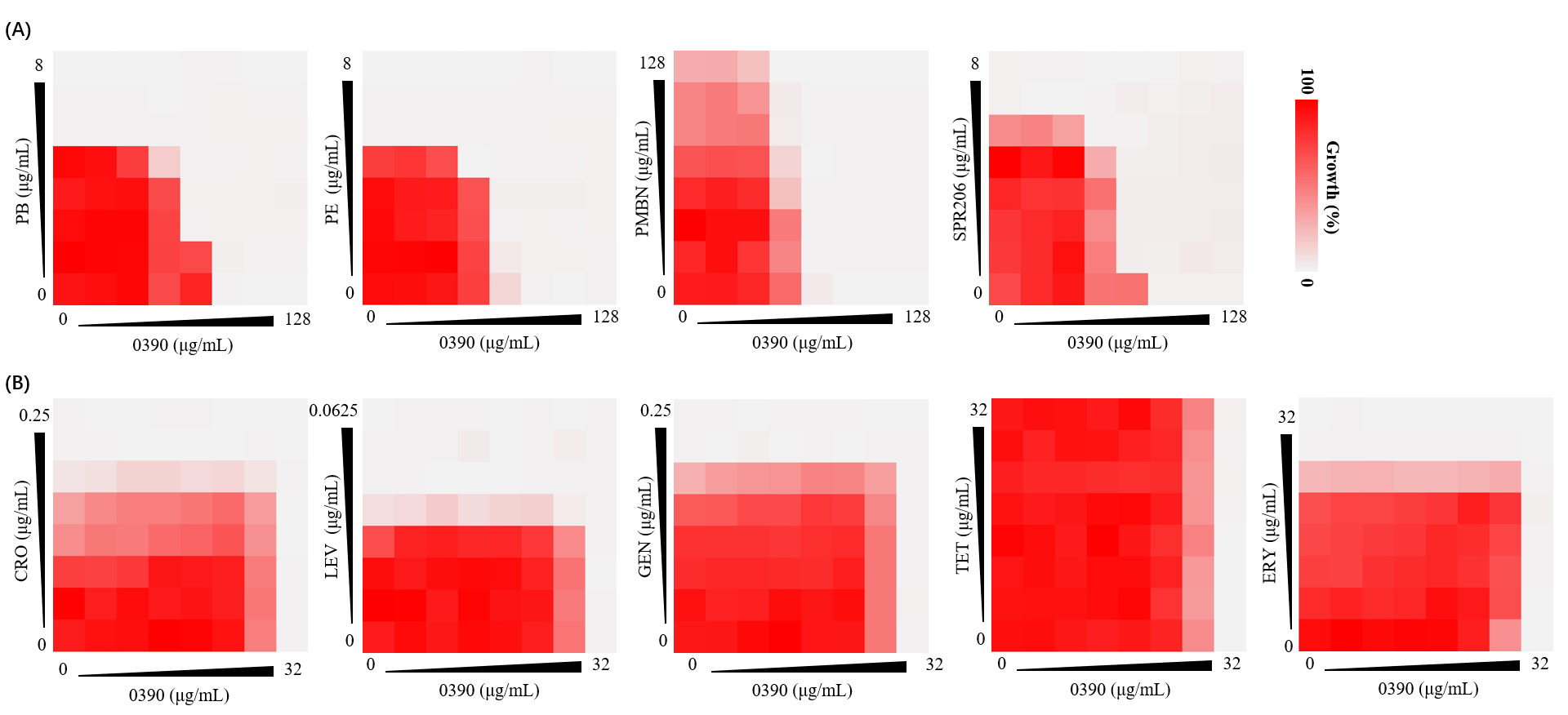

Supplement: Supplementary file 1 [file Data_Sheet_1.zip › Figure S4.jpg]

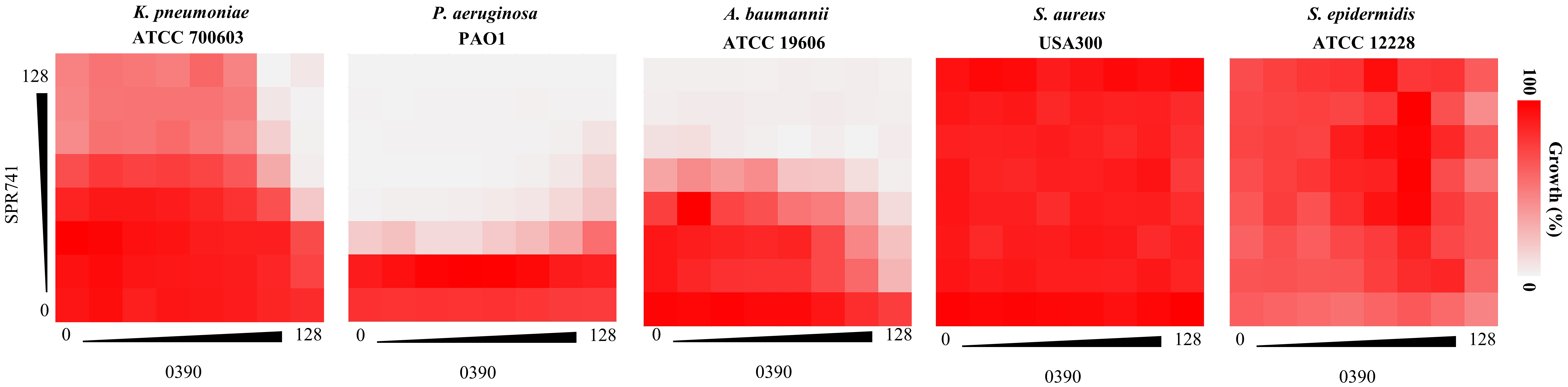

Supplement: Supplementary file 1 [file Data_Sheet_1.zip › Figure S5.TIF]
